# The use of His bundle pacing for the treatment of painful left bundle branch block syndrome

**DOI:** 10.1002/ccr3.2793

**Published:** 2020-03-10

**Authors:** Kevin Andrew Smith, Julie Frey, Amber McKenzie, Kyle Hornsby, John Strobel

**Affiliations:** ^1^ Indiana University School of Medicine Indianapolis Indiana; ^2^ Indiana University Health Southern Indiana Physicians Bloomington Indiana; ^3^ Cardiopulmonary Rehab/Diabetes Center/Advanced Heart Care Center Indiana University Health Bloomington Bloomington Indiana

**Keywords:** bundle of His, chest pain, electrophysiology, His bundle pacing, left bundle branch block

## Abstract

Painful left bundle branch block syndrome is a rare disorder in which patients develop typical angina‐like pain without identifiable ischemia. To date, there have been few published cases of effective treatment. In this case report, we describe successful implementation of His bundle pacing for durable symptom resolution in this disorder.

## Graphical abstract

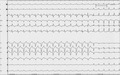

## 1 INTRODUCTION

Painful left bundle branch block (LBBB) syndrome is characterized by the development of intermittent nonischemic chest pain at the onset of exercise‐induced or rate‐related LBBB. It has been postulated that the pain arises from dyssynchronous ventricular contraction via a potential generalized interoceptive sensitivity although the precise mechanisms remain elusive.^1^ Painful LBBB syndrome is a challenge to diagnose, although treadmill EKG testing can often be used to induce LBBB and evaluate concurrent symptoms. In a retrospective study of 9623 patients who underwent treadmill EKG testing,^2^ exercise‐induced LBBB was present in <1% while painful exercise‐induced LBBB was even rarer. Painful LBBB syndrome may be more common than previously reported due to misdiagnosis, reliance on telemetry to detect the LBBB, and termination of treadmill EKG testing if LBBB is detected which may precede the onset of pain.^1^ Treatment options for managing painful LBBB syndrome continue to evolve, and indeed, the literature has very recently begun to display the use of His bundle pacing (HBP) for the treatment of this entity.^3^, ^4^ We report our experience of the successful treatment of painful LBBB syndrome using HBP.

## 2 CASE REPORT

A 49‐year‐old woman with a history of coronary artery disease (CAD) involving stent placement to the mid right coronary artery and mid left anterior descending artery (LAD) in February 2015 and to the proximal LAD in April 2015 continued to have exertional chest discomfort. She had two subsequent coronary angiograms in July 2016 and November 2017 demonstrating patency of stents and mild nonobstructive CAD to a diagonal that was not hemodynamically significant by instant wave‐free ratio. She was given a presumptive diagnosis of microvascular angina and started on ranolazine and continued on treatment with metoprolol. She was referred to cardiopulmonary rehabilitation. She continued to have exertional chest discomfort during cardiopulmonary rehabilitation with corresponding exercise‐induced LBBB (EI‐LBBB) documented via telemetry at heart rates between 100 and 110 bpm. The patient was extremely symptomatic with chest pressure described as a “kick in the chest,” associated with nausea, vomiting, and presyncope in November 2017. Sublingual nitroglycerin provided no relief. Eventually, symptoms resolved within minutes of resolution of the LBBB. Metoprolol succinate was increased to 200 mg daily, and the patient was referred for cardiac electrophysiology consultation noting the correlation in symptoms and appearance of the EI‐LBBB. It was determined that there was sufficient evidence to suspect painful LBBB syndrome, and electrophysiologic testing was recommended to correlate her symptoms with LBBB and to assess symptomatic improvement with pacing. Repeated denials by her insurer for this testing resulted in a 5‐month delay before this evaluation could be completed. During this interval, the patient continued with cardiopulmonary rehabilitation but experienced a significant emotional toll with a markedly decreased quality of life related to time off work and a rapid decline of performing activities of daily living due to continued exertional chest discomfort.

**Figure 1:**
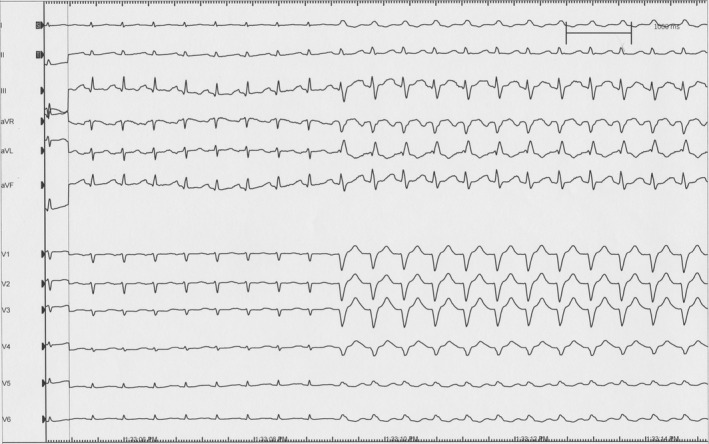
A 12‐lead ECG demonstrates the onset of left bundle branch block following isoproterenol infusion during EP testing is associated with the development of chest pain

**Figure 2:**
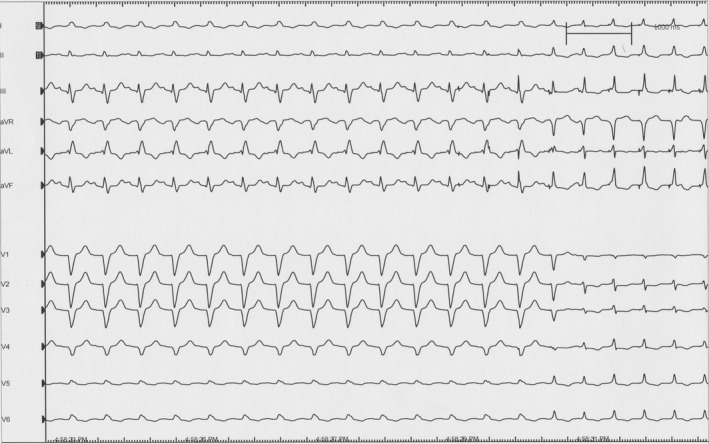
A 12 lead ECG demonstrates the reversal of left bundle branch block by His bundle pacing associated with immediate resolution of chest pain

**Figure 3:**
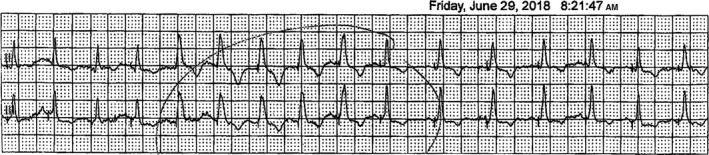
Telemetry of leads II and III showing conversion to left bundle branch block when heart rate exceeds the pacemakers upper tracking rate of 130 bpm. Note the change in morphology congruent with what was seen during the EP study as shown in Figure 1

In June 2018, an EP study was performed with induction of LBBB aberrancy achieved with isoproterenol administration at 4 mcg/min (Figure 1). There was immediate chest discomfort described as a “rolling” followed by pain, confirming the correlation of symptoms with LBBB. His bundle pacing was then performed. With selective His bundle capture, there was resolution of LBBB, and improvement in her symptoms within 10 seconds (Figure 2). The decision was made to pursue implantation of a dual‐chamber pacemaker with His bundle pacing. A dual‐chamber pacemaker was placed, and selective His bundle capture was achieved. Her pacemaker programming was initially set to DDD with a lower rate limit at 60 bpm and upper tracking rate (UTR) of 130 bpm. She was able to walk several laps in the recovery unit while being paced and was entirely symptom‐free prior to discharge the following day. The patient was discharged on metoprolol succinate 50 mg daily. She returned to cardiopulmonary rehab with progressively increased workloads when at her third session, she developed her typical “rolling” symptoms followed by chest pain that took her breath away. This correlated with heart rate exceeding her UTR of 130 bpm resulting in the recurrence of LBBB (Figure 3). Her UTR was increased to 150 bpm without further symptoms. The patient completed her cardiopulmonary rehabilitation in August 2018. She has returned to work and recreational activities and has continued to exercise to heart rates well above 130 bpm without recurrence of symptoms.

## 3 DISCUSSION

Historically, ischemia was hypothesized to be the most likely cause of painful LBBB syndrome, but studies have not supported this. Reviews of the available published reports demonstrate that demand ischemia, coronary lesions, and vasospasm are not the source of painful LBBB syndrome.^1^, ^5^, ^6^, ^7^, ^8^ The current leading hypothesis is that dyssynchronous left ventricular contraction causes the pain as speculated originally by Virtanen et al^9^ and later by Perin et al^10^ This hypothesis is conceptually superior and is consistent with our patient who experienced an immediate “onset/offset” phenomenon of her symptoms coinciding with the development of her LBBB and resolution with His bundle pacing, a pattern that is not typical of angina. Indeed, modern criteria for painful LBBB syndrome generally preclude ischemia as the etiology of the patient's pain. Therefore, this patient's concomitant history of CAD was a confounding factor. She had received adequate treatment, rehabilitation, and follow‐up but continued to be symptomatic. Further investigations revealed no new lesions which could explain this and an EI‐LBBB was discovered. Thus, the diagnosis of painful LBBB syndrome was considered and proven during the EP study.

The usual first attempt at treatment of painful LBBB syndrome is rate control with medications such as beta‐blockers and verapamil in conjunction with cardiac rehab or aerobic exercise, and these were initially attempted in this case. While there has been some evidence of benefit with exercise,^7^ there have been poor results with rate control^8^, ^9^ to the point of worsening symptoms in one case.^10^ With this evidence and the lack of improvement in our patient, we pursued HBP as a novel therapy for painful LBBB syndrome.

His bundle pacing may effectively treat conditions related to bundle branch block since the site of block is often within the His bundle. Predestined fibers in the His‐Purkinje system were first verified experimentally in 1977 by Narula.^11^ From this, it was later discerned anecdotally that most defects causing bundle branch block occur within the His bundle as opposed to distally in the anatomic bundle branches, and further research confirmed that approximately 95% of LBBB is amenable to His bundle pacing.^12^ Despite this knowledge, HBP was not felt to be feasible for routine clinical use for some time. More recently, it has been shown to improve quality of life and cardiac function in patients with entities such as pacing‐induced cardiomyopathy,^13^ AV block, and sinus node dysfunction.^14^

His bundle pacing has also been useful in restoring physiologic conduction and contractility in patients with typical LBBB.^15^ There has been prior documented symptomatic relief of painful LBBB syndrome using cardiac resynchronization therapy (CRT) while another study reported symptomatic relief with implantation of a dual‐chamber pacemaker demonstrating a change in activation pattern as a potential target for treatment.^2^, ^5^ HBP has recently been shown to be an effective alternative to traditional CRT with an LV lead, and indeed may avoid rare sequelae related to electrophysiologic dyssynchrony from biventricular pacing.^16^ In one cross‐over study, HBP was shown to be equivalent to CRT at 6 months, and QRS narrowing was obtained using HBP in 21 of 29 patients (72%) demonstrating BBB.^15^ Given the above evidence, it was postulated that HBP might be an effective therapy for painful LBBB syndrome. To our knowledge, this is one of the first documented uses of HBP in the treatment of painful LBBB syndrome. There are very few publications on the treatment of this condition in general.^1^, ^3^, ^4^ Therefore, this case report is an example of one of the first applications of HBP for a rarely reported condition which it appears suited to treat.

Because painful LBBB syndrome is exceedingly rare, it may be challenging to study HBP as a therapy in a large cohort. On the other hand, painful LBBB may not be as rare as most believe due to a lack of appreciation for this condition, potentially leading to underdiagnosis in many patients. Our application of HBP for painful LBBB syndrome required no special modifications to the typical technique.^17^ The experience with this patient, along with others in recent publications,^3^, ^4^ demonstrates the utility of EP testing in the diagnosis and treatment of this condition. Should you strongly suspect that a patient may have painful LBBB, and other more common and dangerous sources of chest pain have been ruled out, it would be most appropriate to refer them to an electrophysiologist who is comfortable performing HBP. The recommendation of the authors is to perform an EP study using either atrial pacing or isoproterenol to correlate pain with LBBB. Pacing at sites including the His bundle, RV apex, and CS branches to evaluate for symptom resolution could demonstrate the benefit of implanting a permanent pacemaker. For cases in which attempts to correct LBBB through HBP fail, there may be a distal block and we would suggest attempting CRT using BiV pacing or the newer technique of direct left bundle branch pacing if either is feasible.

## 4 CONCLUSIONS

We have described the successful recognition and treatment of painful LBBB syndrome utilizing HBP. Since most conventional therapies which focus on rate control and aerobic exercise have minimal success, we have shown that HBP may be a more promising treatment option, and it deserves continued investigation.

## CONFLICT OF INTEREST

The authors have no conflicts of interest to disclose.

## AUTHOR CONTRIBUTIONS

KS: served as a primary drafter of abstract, introduction, discussion, conclusion, and figures, revised critically, and reviewed literature. JF: involved in concept/design, drafted case report section, and revised critically. AMK: drafted case report section and revised critically. KH: involved in concept/design, implemented the treatment, and revised critically. JS: involved in concept/design and revised critically.
